# NMR Spectroscopy-Based Metabolomics of Platelets to Analyze Brain Tumors

**DOI:** 10.3390/reports4040032

**Published:** 2021-10-01

**Authors:** Shivanand Pudakalakatti, Alessandra Audia, Anirudh Mukhopadhyay, José S. Enriquez, Dontrey Bourgeois, Nabihah Tayob, Niki M. Zacharias, Steven W. Millward, Daniel Carson, Mary C. Farach-Carson, Frederick F. Lang, Amy B. Heimberger, Krishna P. Bhat, Pratip K. Bhattacharya

**Affiliations:** 1 Department of Cancer Systems Imaging, The University of Texas MD Anderson Cancer Center, Houston, TX 77054, USA;; 2 Department of Translational Molecular Pathology, The University of Texas MD Anderson Center Center, Houston, TX 77030, USA;; 3 MD Anderson Cancer Center UTHealth Graduate School of Biomedical Sciences, Houston, TX 77054, USA;; 4 Department of Statistics, Rice University, Houston, TX 77005, USA;; 5 Department of Biostatistics, The University of Texas MD Anderson Cancer Center, Houston, TX 77030, USA;; 6 Department of Urology, The University of Texas MD Anderson Cancer Center, Houston, TX 77030, USA; 7 Department of BioSciences, Rice University, Houston, TX 77005, USA;; 8 Department of Diagnostic and Biomedical Sciences, School of Dentistry, The University of Texas Health Science Center, Houston, TX 77054, USA; 9 Department of Neurosurgery, The University of Texas MD Anderson Cancer Center, Houston, TX 77030, USA

**Keywords:** brain tumor, NMR spectroscopy, platelets, metabolomics, biomarkers

## Abstract

“Tumor-educated platelets” have recently generated substantial interest for the diagnosis of cancer. We hypothesized that tumor educated platelets from patients with brain tumors will reflect altered metabolism compared to platelets from healthy volunteers. Here, in a pilot study, we have employed nuclear magnetic resonance (NMR) spectroscopy in platelets from brain tumor patients to demonstrate altered metabolism compared to the platelets obtained from healthy volunteers.

## 1. Introduction

Brain tumors are a large group of central nervous system cancers that although are not uniformly fatal, can be life changing. Some of them such as gliomas arise in the specialized glial cells and glioblastoma (GBM), a grade IV glioma is a particularly lethal type of brain cancer. The current standard of care for GBM patients includes surgery followed by chemo-radiation. However, these tumors often recur, and early detection of primary and recurrent tumors remains challenging. What often appears to be initial success on post-operative magnetic resonance imaging (MRI), as evidenced by >95% tumor resection, may be followed by tumor recurrence within 3–6 months. An additional challenge for radiologists and oncologists is differentiating between pseudo-progression caused by inflammation and post-radiation edema versus actual disease progression. In other brain cancers, different types of challenges are encountered. Medulloblastomas are childhood brain tumors that are rare, and treatments can cause cognitive dysfunctions. Similarly, ependymomas can occur in any age, and spreads in many regions of the brain and spinal cord. Diagnosis and treatment of brain cancer using nanoparticles has been exploited due to their properties of biocompatibility, biodegradability, surface functionalization, optical, magnetic, and photodynamic properties [1]. The blood-based biomarkers have been explored to assess treatment response and disease status since re-biopsy and repeat surgery may be impractical for brain tumors. However, despite the potential, blood-based biomarkers such as circulating tumor cells, exosomes, and cell free deoxyribonucleic acid (DNA) have yet to become routine clinical diagnostics.

Blood platelets are anucleated cell fragments circulated in the body that originate from megakaryocytes in the bone marrow. Platelets play a vital role in hemostasis and initiation of wound healing [2,3]. Platelets communicate with their surroundings and are activated when they encounter a damaged blood vessel. However, more recently, platelets have been discovered to be involved in a wide repertoire of functions including immune-surveillance and initiating responses to inflammatory diseases to enable tumor growth and metastasis in cancer [4–12]. Platelets release pro-angiogenic and pro-inflammatory factors, which create a favorable environment for tumor growth and play a vital role in the transportation of circulating cancer cells in metastasis. In addition, platelets have been shown to exchange signaling molecules with malignant tumors [13,14]. Recently, peripheral platelet-derived ribonucleic acid (RNA) signatures have been shown to be of diagnostic value for many cancers including grade IV gliomas (glioblastomas or GBMs) [12–14]. Nolte et al. showed a correlation between GBM tumor growth and elevated thrombocytosis [7]. Nilsson et al. have shown the presence of a distinct RNA signature in the platelets of GBM patients relative to a cohort of healthy volunteers [13]. Similarly, platelets are “educated” by tumors, showing the presence of distinct RNA profiles in platelets from 238 pancreatic patients [15]. Several recent reports predicted the presence of non-small cell lung cancer by the analysis of RNA sequencing of platelets using algorithms based on platelet RNA libraries [16]. “Tumor-educated platelets” collected in liquid biopsy have recently generated substantial interest for diagnosis of cancer since platelets will contain a different compilation of RNA in a cancer-free person than tumor-educated platelets in cancer patients. In vitro studies with cell lines also have confirmed the transfer of RNA from cancer cells to platelets. Although the exact mechanisms of this transfer remain unknown, it is thought to involve transfer of microvesicles [13].

Metabolites are typically small molecules that are intermediates or end products of metabolic processes. Metabolite concentrations become altered when there is pathophysiology attributable to a genetic modification, disease, infection, or environmental insult [17]. Variations in metabolite concentrations are a direct readout of the response of a cell, tissue, or an organ to such perturbation. These metabolites can therefore serve as biomarkers for disease or as indicators of therapeutic response including cancer. Although the previously published work has demonstrated correlations between platelets and cancer biology, the current techniques to identify and quantify transcriptomics and proteomics in platelets remain challenging, which limit their practical use. Alternatively, nuclear magnetic resonance spectroscopy (NMR) -based metabolomics profiling of platelets offers a relatively simple, facile, and less expensive alternative [17]. *We hypothesized that platelets isolated from blood of brain cancer patients would exhibit altered metabolism compared to platelets from healthy volunteers*. To test this hypothesis, we chose high resolution NMR spectroscopy as an analytical tool, as it has potential to quantify altered metabolism [18–22]. In this proof-of-concept study, platelets were isolated from healthy donors and donors with brain tumors, and the metabolic activity was profiled.

## 2. Results

**Figure 1.**
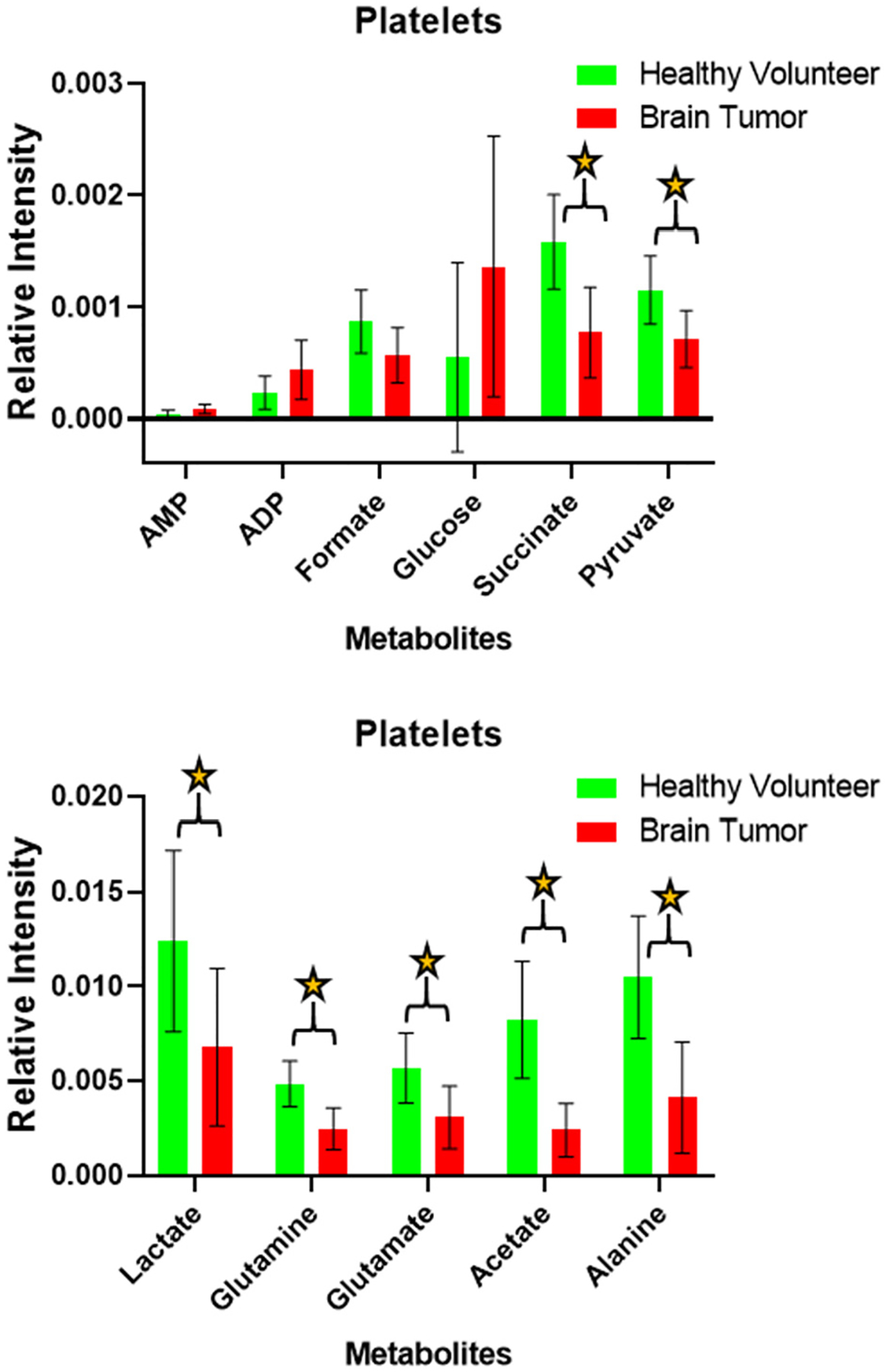
Bar plots of the metabolite’s relative intensities from NMR studies are shown. The relative intensity directly correlates with the concentration of the designated metabolite. The concentrations acetate, alanine, glutamine, glutamate, pyruvate, succinate, and lactate are lower in brain cancer patient platelets (n = 10) compared to healthy volunteers (n = 10). The ‘*’ means *p* value is < 0.01.

Analysis of the 1-D ^1^H-NMR metabolic profiles of healthy volunteer platelets (n = 10) and brain tumor patient platelets (n = 10) revealed that lactate, acetate, glutamine, glutamate, succinate, alanine and pyruvate levels are significantly altered (*p* < 0.01, Figure 1). These data may indicate homeostatic changes of reduced pyruvate, lactate, and alanine in glycolysis and reduced tricarboxylic acid (TCA) cycle activity indicted by reduced concentration of glutamate, glutamine, and succinate in the brain cancer patient-derived platelets relative to normal volunteers. Platelet turnover of adenosine triphosphate (ATP) is faster than in most other cells, indicating a high dependence on energy metabolism for platelet function [23]. The vast majority of ATP provided by metabolic activity is via the TCA cycle and glycolysis, suggesting that platelet function in brain tumor patients is severely compromised. Acetate is another important metabolite, which is significantly reduced in concentration in platelets collected from brain cancer patients, indicating the way fatty acid metabolism is altered. Pyruvate and glutamate can be reversibly produced from alanine and α-ketoglutarate through alanine transaminase (ALT) whenever the pyruvate and glutamate substrates are available. The amino acid glutamine is a key nutrient that takes part in neurotransmission, fuels biosynthetic processes including ATP generation, redox homeostasis, nucleotide, protein, and lipid synthesis [24,25]. The glutamine/glutamate rich microenvironment in which brain tumors grow can induce synaptic connections with glutamatergic neurons and reprogram glutamine metabolism to enable their growth. We may be detecting this rewired metabolism in the platelets. Decreased glutamate can be attributed to cataplerosis through TCA cycle and being used in biosynthesis. These altered glutamate levels are likely to be supported by glutamate transporters [24,26]. However, we did not observe any significant changes in the concentration of glucose between brain tumor and healthy volunteer platelets. Taken together, the mechanisms by which platelets undergo metabolic reprogramming in brain tumor patients remains elusive. Nonetheless, increasing evidence indicates that lower concentrations of lactate, acetate, glutamine, glutamate, succinate, alanine, and pyruvate in brain cancer patients are promising biomarkers.

## 3. Statistics

**Figure 2.**
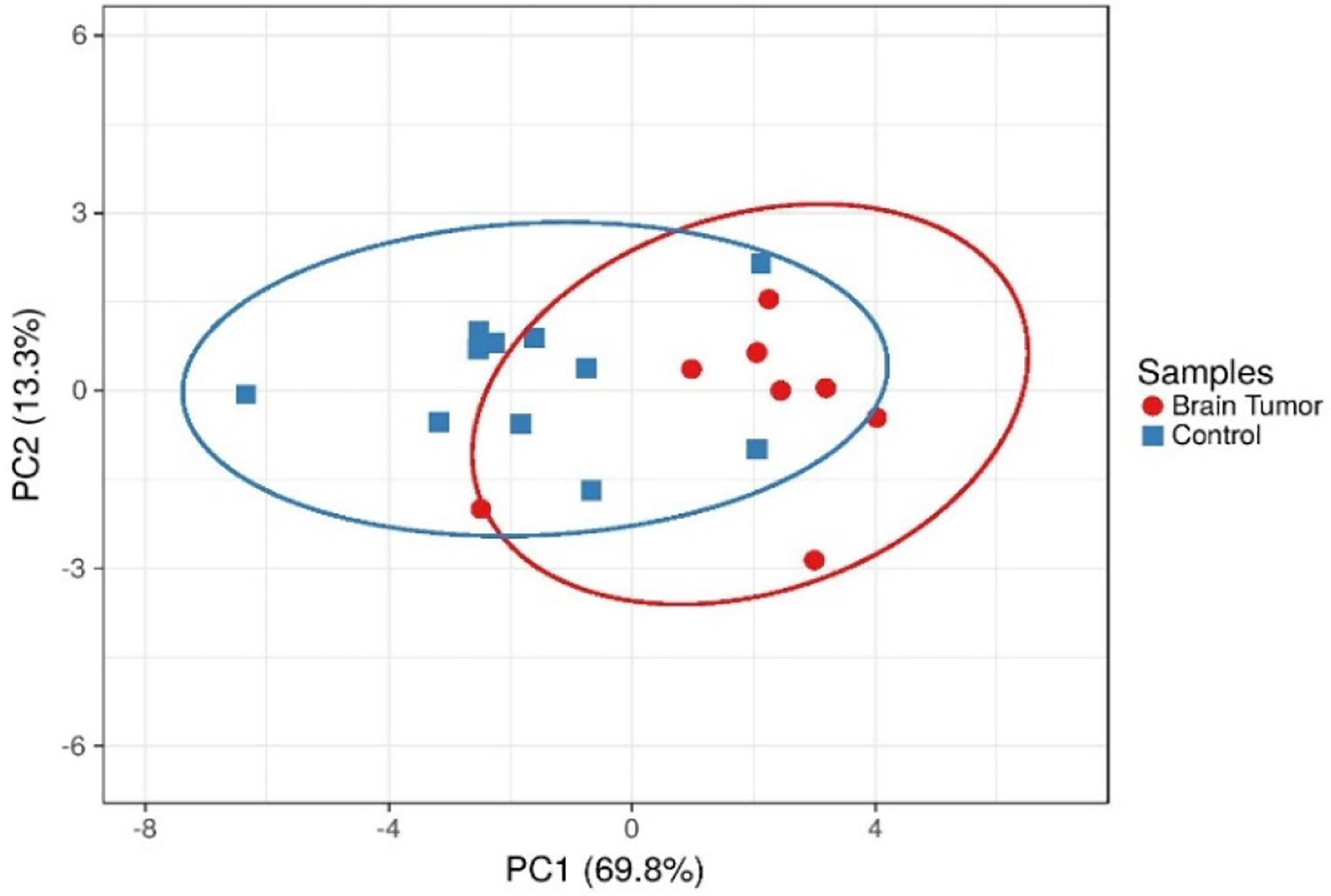
The PC1 and PC2 components together account for more than 85% of variance. The scatter plot shows distinct clustering of data from healthy volunteers versus the brain tumor patients. PCA plots clearly indicate control and brain tumor patient platelets distinctly separated in PC1. PC1 explains 69.8% variance in the data and PC2 explains 13.3% variance in data. Eclipses predict the new observation will fall inside the eclipse with 0.95 probability.

The individual metabolites were compared across patient and healthy volunteer groups using bar graphs, and box and whisker plots. The statistical significance of the observed differences was tested using a multiple unpaired t-test assuming gaussian distribution and each metabolite from both populations have same standard deviation. A principal component analysis (PCA) was used to evaluate whether the metabolomic profiles could be used to separate the patient groups and the healthy volunteers. Glucose, adenosine monophosphate (AMP), adenosine diphosphate (ADP), glutamine, formate, glutamate, lactate, succinate, acetate, pyruvate, and alanine were included in the PCA analysis (Figure 2). The data were generated using a ClustVis: a web tool for visualizing clustering of multivariate data (BETA) [27].

## 4. Discussion

In this study, we detected a metabolic content difference in platelets between healthy volunteers and brain cancer patients using a simple non-invasive method. Analysis of the observed differences in metabolite concentrations between platelets from the latter suggests that platelet-associated lactate, acetate, glutamine, glutamate, succinate, alanine, and pyruvate can be used as a biomarkers for brain cancer. The limitation of our study is that the number samples studied are fewer due to difficulty in obtaining patient platelets. Increasing the number of samples for study will give a very robust biomarker(s) to identify brain cancer patients. This method is analogous to a simple blood test, which is both cost-effective and has high patient tolerance. Future work will focus on comparing these data with other diagnostic techniques, such as magnetic resonance imaging, positron emission tomography, and histology to validate the promise of this technique as a viable clinical tool. Finally, more robust studies should be developed to establish a plausible mechanism that explains the contributory metabolic pathways altered in platelets. These advances may aid personalized medicine and diagnostics.

## 5. Materials and Methods

### 5.1. Blood Collection and Platelet Extraction

The blood and tissues were collected according to institutionally mandated protocol and guidelines for research with signed consent from brain tumor patients. Platelets were collected under a tumor profiling program, known as PROACTIVE (IRB protocol number 2012–0441). The blood was collected the morning one day before the surgery when the patients were not fasting and under a normal diet. Tumors were graded based on the World Health Organization (WHO) classification of central nervous system (CNS) tumors. Isocitrate dehydrogenase-1 (IDH-1) mutational analysis was performed by immunohistoc-hemical (IHC) analyses [28]. The patient population selected for this study is illustrated in Table 1 (brain tumor patient samples). Healthy volunteer blood was obtained from the Institutional Blood Bank using identical protocols as those used for patients.

Blood was collected in BD Vacutainer^®^ purple top tubes (Franklin Lakes, NJ, USA) containing the anticoagulant Ethylenediamine tetraacetic acid (EDTA) to prevent platelets from becoming activated. Platelet rich plasma (PRP) was collected by centrifuging the tubes at a speed of 800 rpm for 15 min. Caution was taken to set the centrifuge to acceleration 5 and deceleration 2 in order to avoid platelet activation. PRP was separated carefully from the other components of the blood. The PRP was centrifuged at 1800 rpm for 10 min after addition of 2 μL of 1 mM prostaglandin−2 to 2 mL of PRP. Of the resulting platelet pellets, one was flash frozen and stored at −80°C, and the other was used for protein quantification. An aliquot of the PRP was collected to estimate the purity of the platelets by staining with an anti-human CD41 antibody (BD Bioscience, USA, cat number #555466) using flow cytometry.

The platelet pellet was lysed in NP40 buffer for 1 h at +4°C in rotation end-over-end. After rotations, the lysate was centrifuged at 8000 rpm for 5 min at +4°C to eliminate the membrane debris. The protein quantification was performed using Bradford assay according to the manufacturer instructions. In this assay, the binding of protein molecules to Coomassie blue dye under acidic conditions results in a color change from brown to blue which absorbance at 565 nm is measured at the spectroscope. The protein concentration is then calculated referring to a standard curve created using various concentrations of bovine serum albumin (BSA).

### 5.2. NMR Data Acquisition

Metabolites were extracted from the platelets using 2:1 methanol–water and ceramic beads as previously described [29,30]. The mixture was vortexed for 40–60 s, snap frozen in liquid nitrogen, thawed on ice, and then repeated for three cycles. This procedure was followed by a 10 min centrifugation at 4000 rpm (3220 × *g*) at 4°C to remove debris. Thereafter, rotary evaporation was used to remove the methanol and overnight lyophilization to remove water. The lyophilized samples were then dissolved in 800 μL of D_2_O and centrifuged at 10,000 rpm for 5 min at room temperature. A 600 μL aliquot of the dissolved sample was added to an NMR tube containing 40 μL of the 8 mM reference compound 4, 4-dimethyl-4-silapentane-1-sulfonic acid-d_6_ (DSS). The final concentration of DSS was 0.5 mM. All supplies were purchased (D_2_O, DSS-d_6_) from Millipore Sigma Aldrich (St. Louis, MO, USA) and used without further purification. All experimental data were acquired using a 500 MHz Bruker Avance III HD NMR equipped with a cryogenically cooled triple resonance (^1^H, ^13^C, ^15^N) Prodigy BBO cyroprobe (Bruker BioSpin MRI GmbH, Ettingen, Germany). Each 1-D ^1^H spectrum was collected with 32K time domain points, 2 s acquisition time, 512 transients, 8012 Hz spectral width, and 6 sec relaxation delay. A 90° pulse of duration 12 μs was used. The water signal was saturated for the length of relaxation time before acquisition of the spectrum [31]. The receiver gain was kept at ~64 for all the spectra. Identification of metabolite peaks was accomplished using the Chenomx NMR Suite (Edmonton, AB, Canada) and the Human Metabolomic Database [32,33]. Finally, the peaks were integrated in Topspin^™^(Bruker) 3.1 and normalized to the DSS reference compound. All 1-D ^1^H NMR spectra were normalized to the platelet protein concentration before analysis.

## Figures and Tables

**Table 1.** Brain Tumor Patient Samples.

Type-Brain Cancer	Sample Code	Sex	Age at Time of Diagnosis	IDH Mutation
Ependymoma	EDTA#1	M	68	Not tested
Gliosarcoma	EDTA#2	F	57	WT
GBM	EDTA#3	M	56	WT
GBM	EDTA#4	M	56	WT
Astrocytoma	EDTA#5	M	56	WT
Medulloblastoma	EDTA#6	M	32	WT
GBM	EDTA#7	M	39	R132C
GBM	EDTA#8	F	50	WT
Meningioma	EDTA#9	F	69	Not tested
Brain metastisis from lung	EDTA#10	M	63	Not tested

IDH = Isocitrate Dehydrogenase mutation, WT = Wild Type, R132C = Arginine 132 to Cysteine mutation.

## Data Availability

The data that support the findings of this study are available from the corresponding author upon reasonable request.
